# Dielectric Strength of Nanofluid-Impregnated Transformer Solid Insulation

**DOI:** 10.3390/nano12234128

**Published:** 2022-11-22

**Authors:** Daniel Pérez-Rosa, Andrés Montero, Belén García, Juan Carlos Burgos

**Affiliations:** Electrical Engineering Department, Universidad Carlos III de Madrid, Leganés, 28911 Madrid, Spain

**Keywords:** transformer cellulose insulation, oil–paper system, nanofluid, nanodielectric, dielectric strength, nanocellulose, breakdown voltage, impulse test, power transformer

## Abstract

The interest in developing new fluids that can be used as dielectric liquids for transformers has driven the research on dielectric nanofluids in the last years. A number of authors have reported promising results on the electrical and thermal properties of dielectric nanofluids. Less attention has been paid to the interaction of these fluids with the cellulose materials that constitute the solid insulation of the transformers. In the present study, the dielectric strength of cellulose insulation is investigated, comparing its behavior when it is impregnated with transformer mineral oil and when it is impregnated with a dielectric nanofluid. The study includes the analysis of the AC breakdown voltage and the impulse breakdown voltage of the samples. Large improvements were observed on the AC breakdown voltages of the specimens impregnated with nanofluids, while the enhancements were lower in the case of the impulse tests. The reasons for the increase in AC breakdown voltage were investigated, considering the dielectric properties of the nanofluids used to impregnate the samples of cellulose. The analysis was completed with a finite element study that revealed the effect of the nanoparticles on the electric field distribution within the test cell, and its role in the observed enhancement.

## 1. Introduction

Transformers are key elements in power systems. Their reliability is basic to ensure a safe and economical supply of electrical energy. In addition, transformer failure may lead to severe accidents which could derive in high economic losses [1,2,3]. One of the main elements that makes the safe operation of transformers possible is the insulation system, which is divided into liquid and solid insulation. Transformer solid insulation is composed of different types of cellulose-based materials such as Kraft paper and pressboard [4]. The liquid insulation is commonly mineral oil (MO), although other fluids are starting to be used, such as natural and synthetic esters.

In the last decades, the use of nanodielectric fluids (NF) has been proposed as an alternative to improve transformer liquid insulation [5,6,7]. An important number of experimental and theoretical works have been reported which support evidence that the addition of small concentrations of nanoparticles (NPs) to insulating oils may have a beneficial effect on the behavior of these fluids [8,9,10]. Enhancement of several oil properties that are relevant in transformer design have been reported, such as AC breakdown voltage [11,12,13,14], impulse breakdown voltage [15,16], and several thermal properties [17,18]. Although the use of these fluids for transformer insulation purposes is far from being feasible nowadays, it is believed that the observed improvements in the properties of insulating oils may lead to the construction of smaller and more efficient devices in the future [19].

The use of NFs as dielectric liquids for use in transformers in service must guarantee that the fluid can remain stable throughout the life of the device and that the interaction of the NFs with other components of the transformer will not cause operational problems.

Few authors have studied the effect of NFs on transformer cellulosic insulation. A few experimental works have analyzed the fundamental behavior of NFs interacting with cellulose materials, finding evidence that, during the impregnation process, a portion of the NPs suspended in the NFs penetrate into the cellulose and interact with this material [20,21,22,23]. The interaction was proved for NFs based on different types of NPs such as TiO${}_{2}$ or Fe${}_{3}$O${}_{4}$. This fact, together with the relevance of the solid insulation for the reliability of the transformer, makes clear the importance of studying in depth how NF-impregnated cellulose behaves.

Some authors have analyzed the dielectric behavior of NF-impregnated cellulose. Most of these studies are related to the characterization of the interface between NFs and cellulose insulation. Rafiq et al. [24] studied the creeping performance of the oil–pressboard interface when an NF based in Al${}_{2}$O${}_{3}$ nanorods was used as insulating liquid; the authors observed an enhancement in the creep voltage with respect to that obtained when using MO. Huang et al. [25] tested the oil–pressboard interface under impulse using TiO${}_{2}$ NPs to manufacture the NF. The study showed important improvements of the flashover voltage, which reached 30% when NPs of diameter 5 nm were used; for larger NP sizes, the improvements were smaller. Another study was performed by Shan et al. [26], in which the focus was placed on the creep phenomena under lighting impulse; they observed a reduction in creepage probability for the same voltage for NF-impregnated pressboard. Maharana et al. [27] studied the AC breakdown voltage (BDV) of pressboard impregnated with NFs. They compared the dielectric strength of NF-impregnated pressboard under several aging conditions with the performance of MO-impregnated pressboard with the same aging degree, concluding that for fresh samples, the improvement in BDV is about 2%. Another interesting study was performed by Shan et al. [28], who studied the effect of the NPs’ diameter on the DC BDV of NF-impregnated Kraft paper; the authors reported a strong decrease in the breakdown strength as the NP size increases. Liao et al. [29] studied the influence of the concentration of NP in TiO${}_{2}$ NP-modified pressboard, observing an increase in the dielectric strength of the modified pressboard. They also observed an important influence of the NPs’ concentration on the dielectric strength and for samples with high NP concentration (i.e., above 3 wt%), the samples started to show a decrease of the dielectric strength.

Table 1 summarizes the previous works published to date on the topic of dielectric properties of NF-based cellulose insulation. Details about the materials used in the study, the tests carried out, and the results are included in the table.

The aim of the present work is to obtain insight into the dielectric performance of NF–cellulose insulation systems. Since most of the authors focused their studies on the oil–cellulose interface, the study of the dielectric strength of impregnated Kraft paper should be performed to better understand the behavior of the material under real operating conditions. For this purpose, the breakdown voltage of Kraft paper samples impregnated with a Fe${}_{3}$O${}_{4}$-based NF was tested under AC voltages at power frequency and under lightning impulses. The tests were repeated with samples impregnated with MO to study the impact of the NP on the dielectric strength of oil-impregnated solid insulation.

The choice of Fe${}_{3}$O${}_{4}$-based NFs was motivated by the good dielectric performance of these liquids, which were investigated in the past by the authors. Improvements of up to 30% were observed on the dielectric strength of the base fluids when small concentrations of Fe${}_{3}$O${}_{4}$ NPs were dispersed in them. Testing the impact of applying these liquids to impregnate the cellulose is important to assess the suitability of those NFs as liquid insulation for transformers.

## 2. Materials and Preparation of the Samples

As was explained before, the main aim of the present study is to analyze the dielectric behavior of Kraft paper samples impregnated with several insulating fluids, and to compare the AC and impulse dielectric strength results obtained on NF-impregnated paper samples with those obtained for MO-impregnated specimens. This section provides information on the materials used for the experimental study and describes the sample preparation process.

### 2.1. Cellulose Insulation

The cellulosic material tested in this work was transformer Kraft paper with grammage 0.75 g/cm${}^{3}$, thickness 0.085 mm, and thermal class 105 ${}^{{}^{\circ}}$C. The typical composition of this type of cellulosic insulation is 75–85% alpha cellulose, 10–20% hemicellulose, and 2–6% lignin [30].

Kraft paper sheets were cut into squares of dimension 8 × 8 cm. The samples were subjected to drying under vacuum at 70 ${}^{{}^{\circ}}$C for 24 h. A relatively low drying temperature was chosen to avoid thermal aging of the samples. However, given the small thickness of the paper, the moisture content after drying was below 1%. After that, as will be explained below, some of the samples were impregnated with MO and some others with NF to compare the dielectric behavior of the two types of composites.

### 2.2. Preparation of the Nanofluids

The preparation and the stability study of the NFs used in this study are fully described in a previous publication [31].

Commercial MO Nytro 4000X (Nynas AB, Stockholm, Sweden) was used in this work to impregnate a part of the Kraft paper samples and it was also used as base fluid in which we immersed the other part of the Kraft paper samples.

The NFs were manufactured by dispersing Fe${}_{3}$O${}_{4}$ NPs in the MO. The Fe${}_{3}$O${}_{4}$ NPs were supplied by the company Magnacol (Newton, United Kingdom) in form of an NP dispersion which used the MO Nytro 4000X as dispersant.

The NFs were prepared in a two-step process by mixing the base MO with the Fe${}_{3}$O${}_{4}$ dispersion to a concentration of NPs 0.2 g/L. This concentration value was chosen because in previous studies the NFs with that concentration of NPs showed the highest AC BDV values, while for higher concentrations, the AC BDV of the liquid decreased [12].

The dispersion of the NPs was performed using an ultrasound stirrer (Inc. model VC 750 W, manufactured by Sonics & Materials (Newtown, CT, USA)) with ultrasound wave intensity 268 W/cm${}^{2}$ for two hours in intervals of 30 s of agitation and 30 s of pause to avoid overheating of the mixture.

The long-term stability of the resulting colloids was evaluated in depth using visual inspection and particle-size measurement with dynamic light scattering. The results of the stability study are reported in detail in [31]. The stability was tested at room temperature and in a temperature range between 25 and 80 ${}^{{}^{\circ}}$C. At room temperature, the NFs remained stable for more than ten months, with diameters of the NPs dispersed in the fluids ranging between 10 and 15 nm. Regarding the stability at higher temperatures, the NFs remained stable for at least two months at temperatures below 60 ${}^{{}^{\circ}}$C; however, the stability was compromised for working temperatures above 60 ${}^{{}^{\circ}}$C. To avoid stability problems, only temperatures below 60${}^{{}^{\circ}}$ were considered in this work. The influence of the presence of paper in the stability of the fluid was also tested, observing no influence from this factor.

### 2.3. Impregnation of Cellulose Samples

Before impregnation of the paper samples, NF and MO were dried under vacuum.

**Figure 1:**
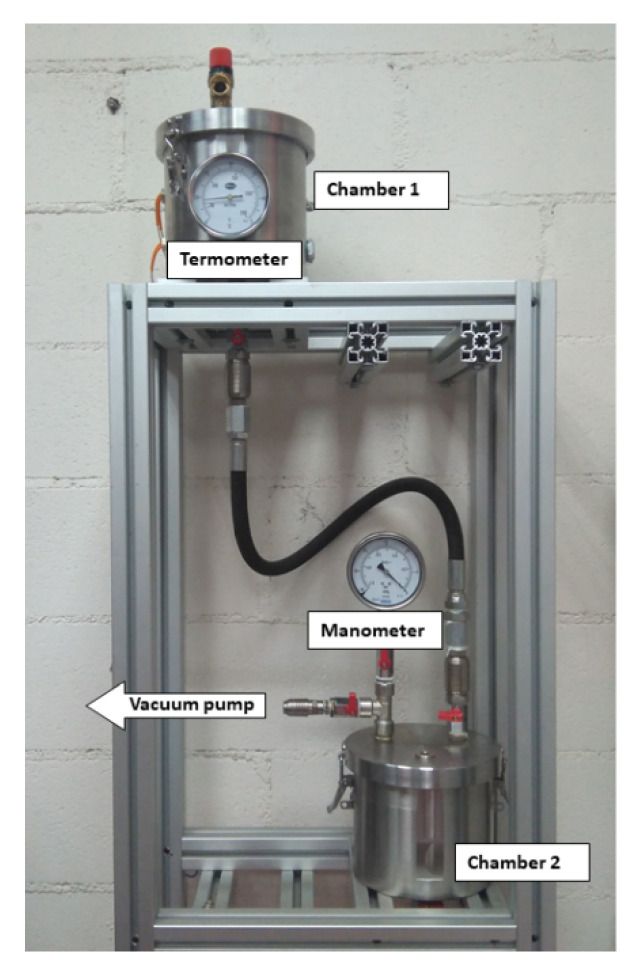
Oil impregnation plant.

After the conditioning process, the paper was impregnated in the impregnation plant shown in Figure 1, which emulates the impregnation process applied in a transformer factory.

**Figure 2:**
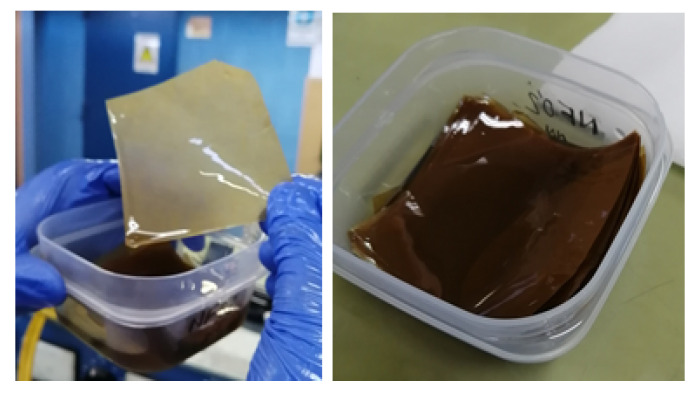
Oil-impregnated paper samples.

For the impregnation, the Kraft paper samples were placed in the lower chamber of the plant, which was subjected to vacuum. The upper chamber was filled with the dielectric fluid used to carry out the impregnation and it was preheated to 50 ${}^{{}^{\circ}}$C. Then, the hosepipe that connect both chambers was opened and the vacuum in chamber 2 caused the fluid to flow as a spray. When chamber 2 was completely filled with dielectric fluid, the system was kept under vacuum for 30 min to ensure proper impregnation of the samples. After that, the dielectric fluid was drained through a valve at the bottom of chamber 2 and the samples were removed and stored immersed in oil or NF until the moment of dielectric strength measurements (Figure 2). The time between the preparation of the NF, the impregnation of paper, and the dielectric strength measurements was less than one week.

**Figure 3:**
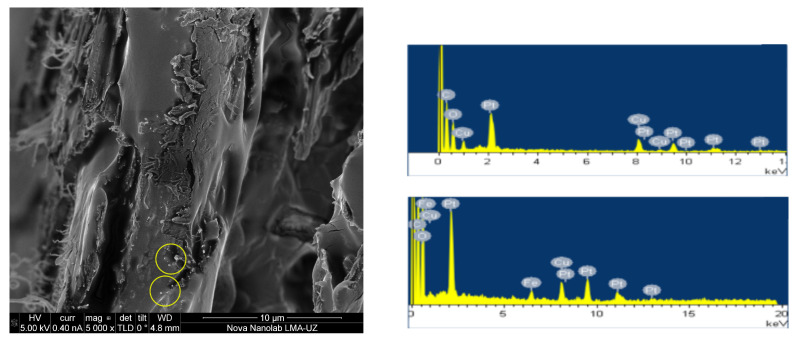
**Left**: Cryo-SEM image of the NF-impregnated Kraft paper (yellow circles mark images identified as NPs). **Right**: EDX spectra of an MO-impregnated paper (**top**) and NF-impregnated paper (**bottom**).

The morphology of the NF-impregnated paper was evaluated by the authors in a previous work using a cryo-scanning electron microscope (Cryo-SEM), energy-dispersive X-ray spectroscopy (EDX), and Fourier-transform infrared spectroscopy (FTIR) [22]. That study revealed that some of the NPs suspended in the impregnating fluid penetrate into the paper and bind to the cellulose molecules. A cryo-SEM image of a cross-section of NF-impregnated paper is shown in Figure 3. In the image there are some bright spots (marked with yellow circles in the figure) that are probably NPs deposited in the cellulose structure. The presence of iron in the paper impregnated with NF was also evidenced by EDX tests. The right part of Figure 3 compares the spectra obtained on an MO-impregnated paper sample (top), which showed no presence of iron, with that obtained on an NF-impregnated paper sample (bottom) where iron was identified. The whole study is reported in detail in [22].

## 3. Dielectric Strength in AC

### 3.1. Test Setup

**Figure 4:**
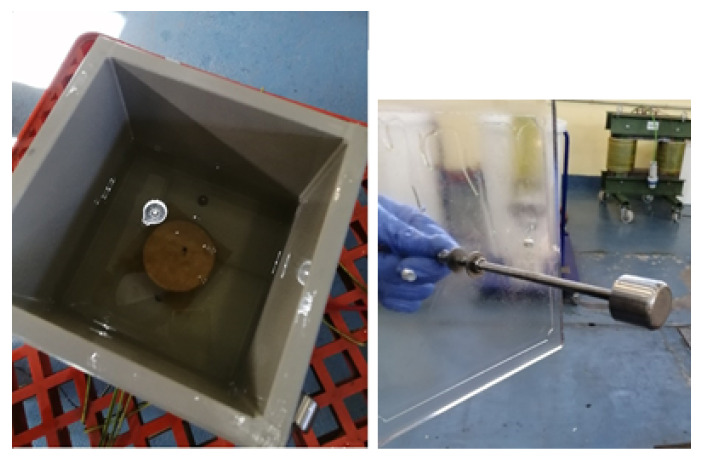
Test cell used in the study.

The MO-impregnated and NF-impregnated samples were tested under AC power frequency voltages (50 Hz). The tests were carried out in an accredited laboratory according to Standard IEC 60243-1 [32], which is the standard recommended in IEC 60641-2 to characterize the dielectric strength of pressboard and paper samples [33]. The cell shown in Figure 4 was used during the tests. The cell is made of a plastic material and has the shape of a cube with dimensions 20 × 20 × 20 cm. The electrode system was parallel-plate with unequal electrodes; stainless steel cylindrical electrodes of diameter 25 and 75 mm were used as stated in IEC 60243-1 [32].

**Figure 5:**
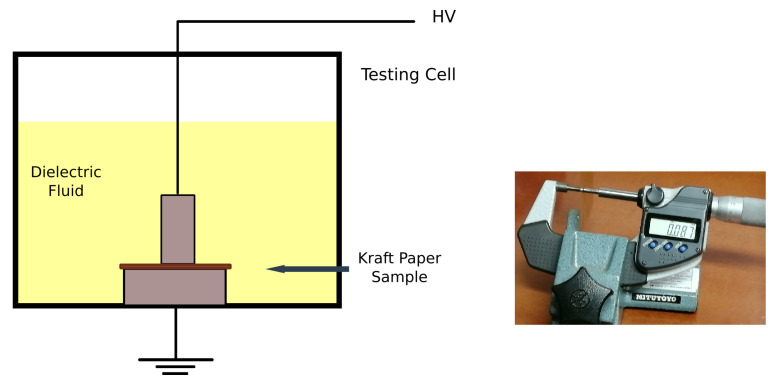
Test cell with Kraft paper specimens surrounded by oil. Thickness measurement.

**Figure 6:**
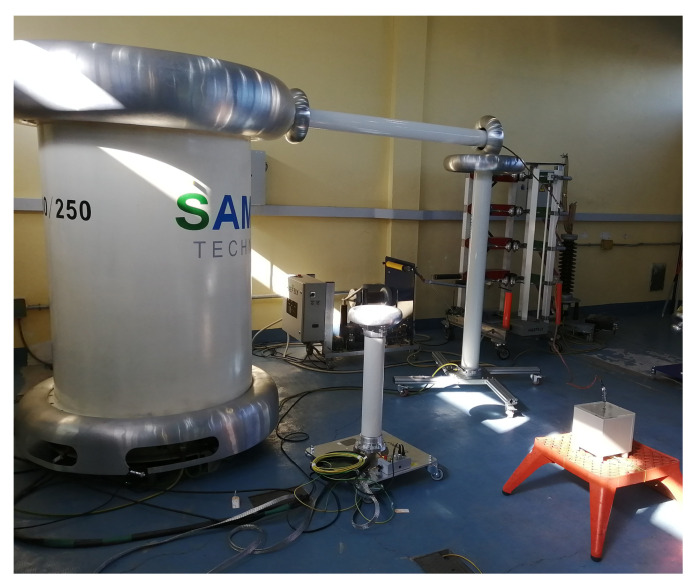
Step-up transformer and test cell for the AC dielectric strength tests.

The measuring process is as follows: A paper sample is placed between the electrodes of the test cell. The test cell is filled with the dielectric fluid until the electrodes are fully covered. The bottom electrode is grounded and AC voltage is applied to the top electrode (Figure 5). The test voltage is supplied by a step-up transformer (Figure 6). The voltage is increased according to the specification of a rapid-rise test, i.e., the applied voltage is increased from zero until breakdown occurs at a rate of 1500 V/s. The choice of rapid-rise test was motivated by the small thickness of the paper samples. After breakdown, the paper sample is removed from the cell and its thickness is measured four times with accuracy $\pm 1.10^{-3}$ mm. The average thickness obtained during the tests was 0.0852 mm, and the standard deviation of the thickness measurements was 0.0046.

The AC dielectric strength (kV/mm) of each specimen is obtained as the ratio between the voltage at breakdown and the average thickness of the sample. Then, a new sample is placed in the cell and the procedure is repeated.

To determine the AC dielectric strength of each material, nine NF-impregnated samples and nine MO-impregnated samples were tested.

### 3.2. Results of AC Dielectric Strength Tests

The mean values obtained for the two types of samples were 75.3 ± 0.7 kV/mm for the NF-impregnated samples and 59.8 ± 0.4 kV/mm for those impregnated with MO. This suggests an improvement of the AC dielectric strength of 26%. The standard deviation of the measurements decreases from 11.24 for the MO-impregnated samples to 5.47 for the NF-impregnated ones.

The experimental data were fitted to a Weibull distribution (Equation (1)), which provides the failure probability associated with a certain voltage level.

(1) $$P\left(E\right)=1-e^{{\left(\frac{E}{a}\right)}^{b}}$$

where P(E) is the failure probability of a test specimen when subjected to an electric field *E*, *a* is the scale parameter (which is related to the field for breakdown probability 63%), and *b* is the shape parameter (inversely related with the scattering of the data).

The parameters obtained for each material are shown in Table 2. As can be observed, the values of the parameters confirm the statistical results. The shape parameter (*a*) reveals an increase in the dielectric strength of the NF-impregnated samples compared to those impregnated with MO. In addition, the shape parameter (*b*) confirms that the measurements are less scattered when NF is used as impregnating fluid.

**Figure 7:**
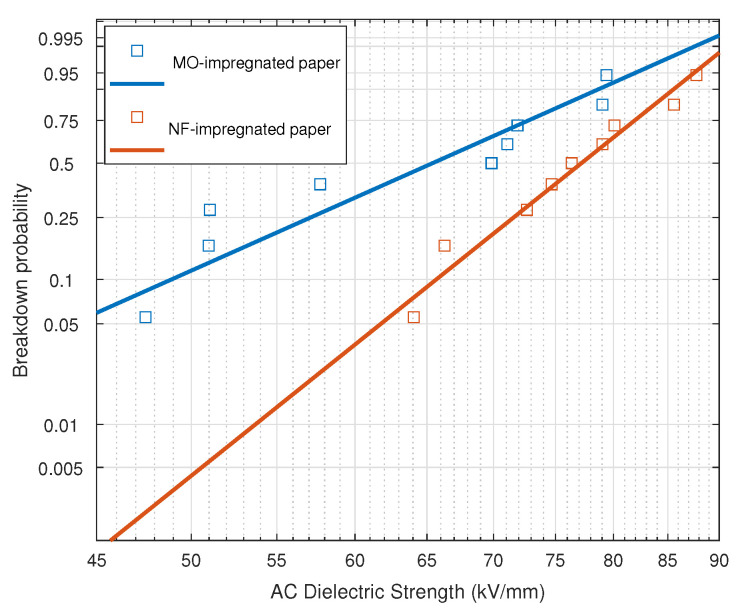
Weibull distribution of the AC dielectric strength measures on NF-impregnated paper and MO-impregnated paper.

The plotting of the Weibull distribution obtained for the NF- and MO-impregnated test specimens is shown in Figure 7.

As can be seen, the failure probability of both materials at a certain voltage is quite different. For the MO-impregnated specimens, the lower quartile of the samples has a dielectric strength between 45–55 kV/mm, while in the NF test specimens, only 1% has a dielectric strength in that range. For any value of the field, the probability of failure in the MO-impregnated paper is greater than the probability of failure in the NF-impregnated paper, although as the electric field increases, the difference between the AC dielectric strength of the samples impregnated with each of the fluids tends to decrease. The main values obtained from the Weibull distribution are shown in Table 3.

### 3.3. Discussion

It was shown that the AC dielectric strength of cellulose specimens increases by 26% when Fe${}_{3}$O${}_{4}$ NPs are added to the impregnating liquid.

The observed improvement could be partially due to the enhancement of the dielectric properties of the MO when the NPs are added. In a previous work [12], the authors investigated the dielectric properties of the NF used for the impregnation of the samples of the present study. The measures revealed that the AC breakdown voltage of the Fe${}_{3}$O${}_{4}$-based NF with NP concentration of 0.2 g/L was only 10% higher than the AC breakdown voltage of the MO (i.e., 95.21 kV vs. 87.02 kV).

Additionally, in a previous work [34], the authors characterized the change in the permittivity of MO when NPs were added to it and also the variation of the permittivity of paper when it was impregnated with NF. The measurements were performed with the dielectric response analyzer Dirana (Omicron). The permittivities of both materials increased significantly, which may be related to the polar character of Fe${}_{3}$O${}_{4}$ NPs. In the case of the liquids, the permittivity changed from 2.2 for MO to 3.2 for NF, while for the MO-impregnated and NF-impregnated paper, the permittivity changed from 3.9 to 4.9. The change in permittivities will lead to a variation of the electric field distribution within the test cell, which can be a factor of influence in the enhancement of the dielectric strength of NF-impregnated paper.

In order to better understand the impact of the permittivity change in the AC dielectric strength tests, a finite element study was performed using COMSOL Multiphysics. An axisymmetric 2D model was built considering the geometry of the test cell and the electrodes, and the permittivity of the materials. The simulation was repeated considering the relative permittivities obtained experimentally for each combination of materials (i.e., MO and MO-impregnated paper vs. NF and NF-impregnated paper). The permittivities considered for the simulations are shown in Table 4.

The testing voltage considered for both simulations was 5.5 kV, which is the average testing voltage at which the breakdown of MO-impregnated paper samples takes place. The same voltage was considered for the NF-impregnated samples, aiming at comparing the effect in the same testing conditions of the two combinations of materials.

**Figure 8:**
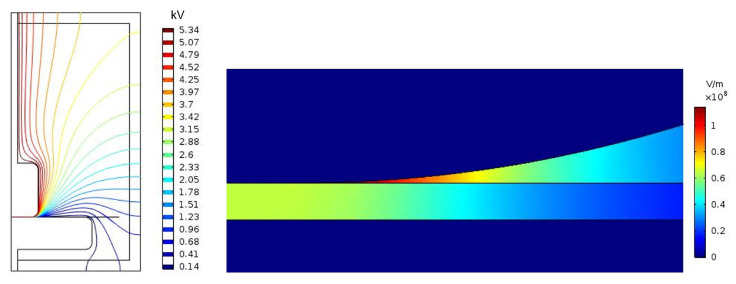
Finite element simulation of the test cell for the MO-impregnated paper immersed in MO.

**Figure 9:**
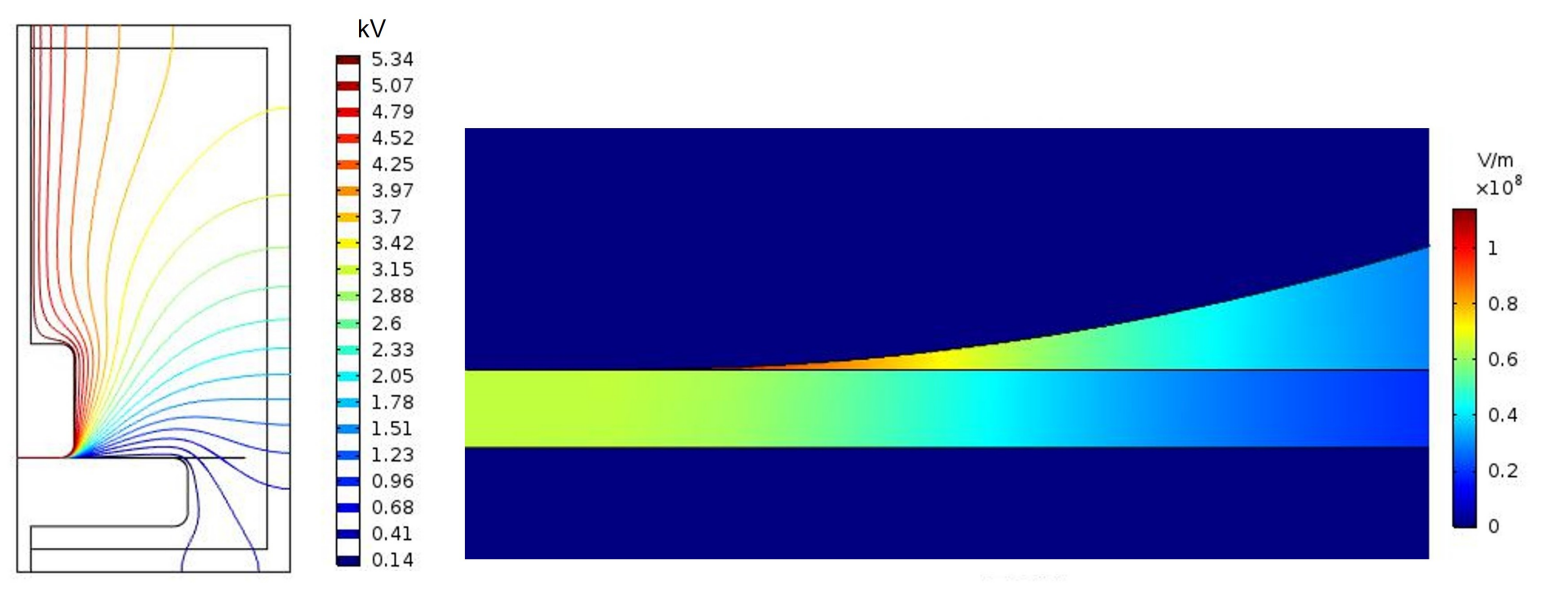
Finite element simulation of the test cell for the NF-impregnated paper immersed in NF.

Figure 8 and Figure 9 compare the results of the finite element simulations for the MO and the MO-impregnated paper specimen (Figure 8) and for the NF and NF-impregnated paper specimen (Figure 9). Both figures show the electric potential lines in the cell and the electric field in the region of maximum stress.

In the MO-impregnated samples, the potential lines converge at the boundary between the upper electrode and the paper (Figure 8). This is also the region that withstands the maximum electric field, as can be observed in the detail provided at the bottom of the figure. In particular, the most stressed area of the whole cell is the volume of oil in that region. It is also interesting to see that there is a large electric field gradient between the oil and the paper.

The NF-impregnated samples present a similar distribution of electric potential lines to the MO-impregnated samples (Figure 9), and the difference between the two cases is not noticeable at first sight. However, the representation of the electric field in the bottom image reveals differences in the distribution of the electric field when compared with Figure 8. In the case of the NF-impregnated samples, the electric field is lower at the point of contact of the upper electrode and the paper sample than in the case of the MO-impregnated samples. Additionally, the electric field is less concentrated in that point and the field gradient between oil and paper is significantly smaller than in the previous case.

The value of the maximum electric field (E) in the oil and in the paper obtained for each simulation are shown in Table 4. As can be seen, the value of the maximum electric field in the paper samples for both combinations of materials is almost the same in the two cases, but the maximum electric field in the liquid is 15% lower in the case of the NF and NF-impregnated paper specimens. Additionally, the maximum electric field in the NF-impregnated paper immersed in NF was recalculated for an applied voltage of 6.4 kV, which is the average voltage at which breakdown takes place in this combination of materials. The simulation calculated that the maximum electric field for the oil was 116.35 kV/mm, while the maximum field for the paper was 75.99 kV/mm.

The simulation results suggest that the breakdown process starts in the fluid. The ionization of the fluid would start in the region of maximum stress and the streamer progresses through the fluid until it reaches the paper and causes its perforation.

**Figure 10:**
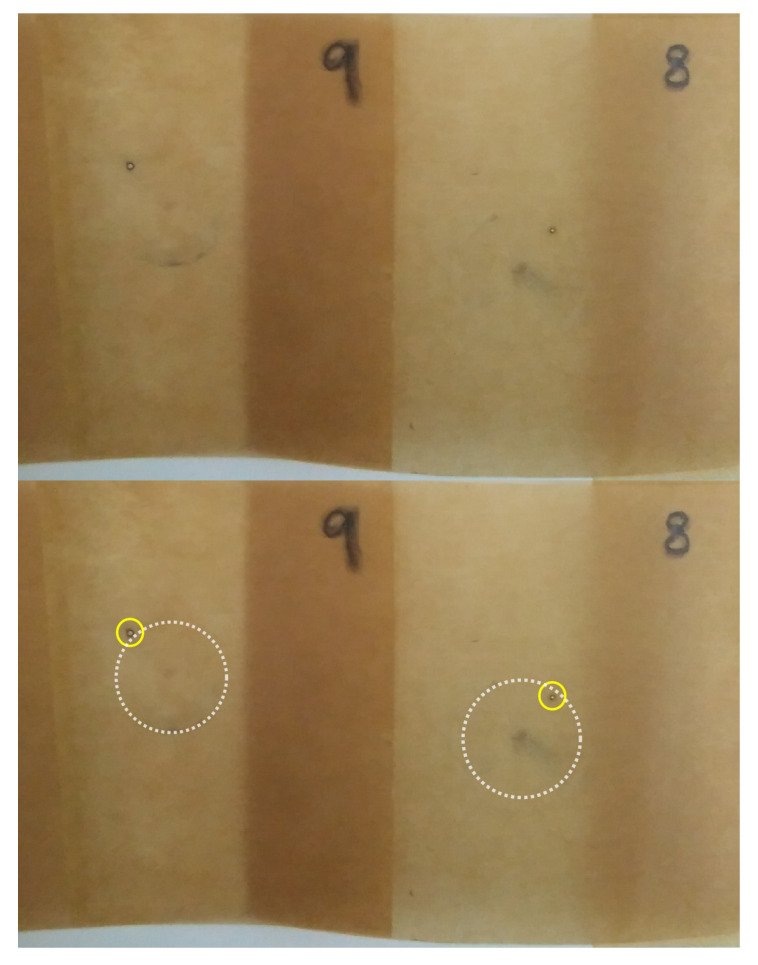
Image of two samples after the breakdown.

This mechanism is in agreement with the visual inspection of the samples. Figure 10 shows an image of two of the paper samples after the breakdown test. The breakdown point and the mark of the upper electrode on the paper surface can be appreciated in the upper image; the breakdown point and the shape of the upper electrode are highlighted in the bottom figure for better identification. As can be seen, the breakdown point is located at the limit of the upper electrode which, according to the simulations, is the region where the maximum electric field is obtained. The same situation was observed in most of the tested samples, since they showed perforation at the edge of the top electrode.

The conclusion of the above analysis is that the observed improvement of the AC dielectric strength in the NF-impregnated samples is mainly due to two phenomena:

1. The decrease in the maximum electric field in the fluid, caused by the change in the permittivity of the materials, reduces the probability of streamer inception. Note that the maximum electric field in the NF was 15% smaller than the maximum field in the most stressed area of the MO.
2. The presence of NPs in the NF hinders the streamer progression in it. This phenomenon was studied by several authors who attributed it to the accumulation of charges around the NPs [35] and to the reduction of the streamer speed, by the trapping and detrapping of charges in shallow traps, which increases sharply when MO is doped with NPs [36]. The improvement of the AC dielectric strength of the NF used in this work is 10%, according to previous works of the authors [12].

The interface polarization or the interface electric field between oil and paper might be an important factor affecting the breakdown strength, in addition to the change on the macroscopic field.

## 4. Lightning Impulse Tests

### 4.1. Testing Procedure

In order to complete the analysis of the dielectric performance of the NF-impregnated paper, MO- and NF-impregnated paper samples were tested under positive lightning impulse (1.2/50 µs). Positive impulse tests were chosen since they pose a higher risk for the insulation, and also because greater improvements were reported for the positive impulse dielectric strength of oils when NPs were added to them.

Impulse tests were carried out using the same test cell and electrodes described in the previous section and shown in Figure 4.

Impulse tests were performed in accordance with IEC 60243-3 [37] using an impulse generator Haefely Type P35 and an oscilloscope Tektronix TDS 744A. The testing procedure is as follows. A paper sample is located between the electrodes of the test cell, which is filled with the insulating fluid. A first impulse is applied with peak voltage value, 70% of the expected breakdown value. In this case, the initial impulses were of value 7 kV. If no breakdown takes place, two more impulses of the same voltage are applied. After applying three impulses without breakdown, the voltage is raised in steps of 1 kV and three new impulses are applied until the breakdown happens. After a breakdown occurs, the sample is extracted from the cell and four thickness measurements are carried out to obtain the average thickness of the solid sample. The impulse strength of the sample is obtained as the ratio between the voltage at which the breakdown occurred and the average thickness of the sample. Finally, a fresh sample is placed in the testing cell and the same measuring sequence is repeated. For the lightning impulse study, eight MO-impregnated paper samples and six NF-impregnated samples were tested.

### 4.2. Impulse Dielectric Strength Results

Table 5 and Table 6 present the total number of impulses applied on each type of sample and the number of breakdowns recorded for each voltage level. As can be seen, all the samples withstood the impulses with a voltage lower than 8 kV. For testing voltages above 9 kV, a variable number of breakdowns occurred in both types of samples. The value of the impulse strength of each sample is calculated as the ratio between the breakdown voltage and the average thickness of the sample. The average value of the impulse strength of MO-impregnated paper immersed in MO was 121.8 ± 0.8 kV/mm, and the impulse dielectric strength of NF-impregnated paper immersed in NF was 124.1 ± 0.8 kV/mm.

**Figure 11:**
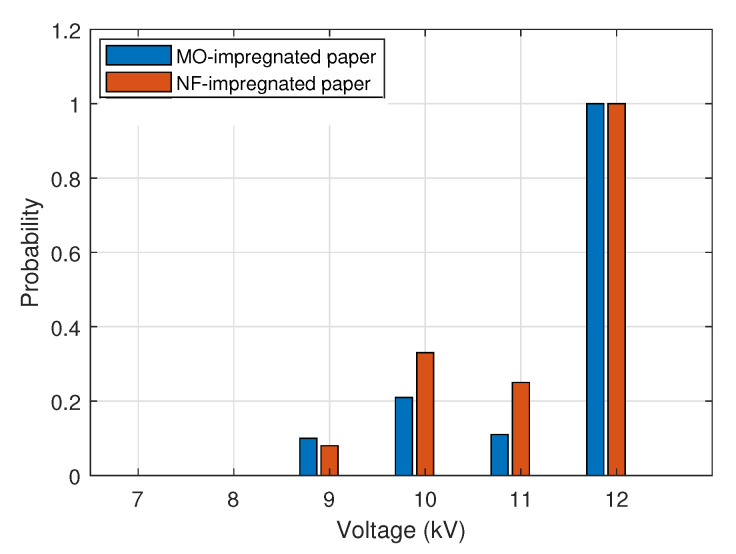
Probability of breakdown in impulse tests.

Figure 11 shows the probability of breakdown for each voltage level for the MO-impregnated and the NF-impregnated samples. It can be seen that for both impregnating fluids the probability of breakdown is zero for voltages 7 and 8 kV. For higher voltage levels, the probability of both fluids is quite similar, and for voltage 12 kV the probability of breakdown is 100% for the two types of samples.

**Figure 12:**
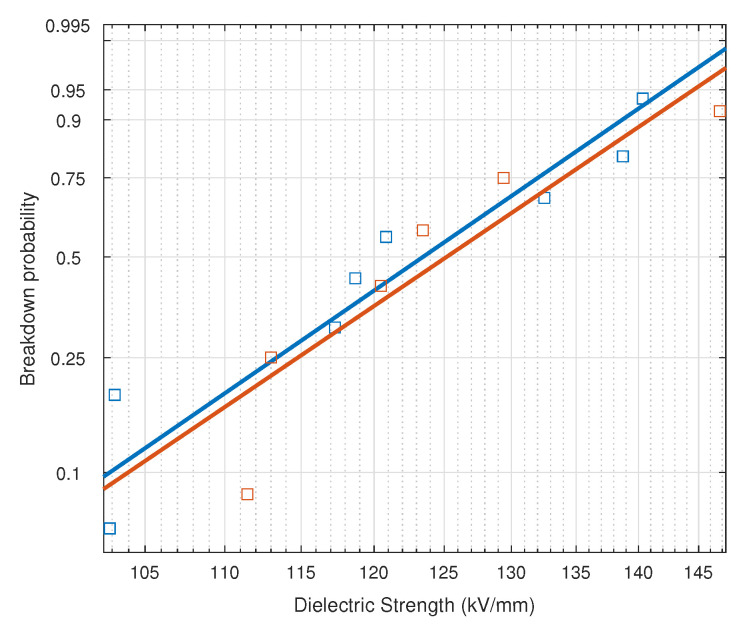
Weibull distribution of the impulse measures on NF-impregnated paper and MO-impregnated paper.

As in the previous study, the experimental data were fitted to a Weibull distribution. The parameters of the Weibull fitting for the impulse test data are shown in Table 7; it can be noted that the scale parameter is about 2 kV/mm higher for NF-impregnated samples, and the shape parameter is almost equal for both cases. The representation of the Weibull distribution for both materials, shown in Figure 12, also reveals a very similar behavior for both kinds of samples.

The main values obtained from the Weibull distribution for the impulse test are summarized in Table 8. The analysis reveals a small improvement of the dielectric strength of the NF-impregnated samples under positive lighting impulse. The mean value of the impulse strength for the NF-impregnated samples is only 2% higher than that of the MO-impregnated samples. The values for the different failure probabilities of both liquids are also very similar.

### 4.3. Discussion on Impulse

Although the NF-impregnated papers present a slightly higher impulse dielectric strength than the MO-impregnated samples, the difference is much lower than for the AC dielectric strength tests.

In a previous work [15], the same NFs used for this study were subjected to impulse tests, considering different NP concentrations. Although the processes involved in the breakdown of paper and oil are probably different, it is worth mentioning that for NP concentration of 0.2 g/L, the impulse BDV of the liquids did not show a clear improvement either, while for higher concentrations, relevant improvements were observed (Table 9). It would be interesting to repeat the impulse study on paper samples impregnated with NFs prepared with higher NP concentrations in the future.

The effect of the change in the permittivity of the materials on the electric field distribution is not easy to evaluate by simulation in this case because of the variation of the high-frequency permittivity parameters and other issues related to the numerical simulation of very fast phenomena. However, an additional round of impulse measurements was carried out in which the test cell was filled with MO and the test specimens were paper samples impregnated with NF. The average impulse dielectric strength in this case was 123.9 ± 1 kV/mm, which is between the results of the two types of samples analyzed in this work.

## 5. Conclusions

Although a number of authors have demonstrated that the dispersion of small amounts of NPs in dielectric fluids improves the dielectric strength of these liquids, the effect of the NPs on other insulating materials that may be in contact with fluids has not yet been documented. In the case of NF-based transformers, the performance of paper and pressboard in the presence of NPs would be especially critical. The present work evaluates the modification of the dielectric strength of samples of transformer Kraft paper when it is impregnated with a Fe${}_{3}$O${}_{4}$-based NF.

The tests carried out show an improvement of 26% on the AC dielectric strength values of the NF-impregnated samples. The authors propose that the observed improvement is due to the effect of the NPs on the dielectric performance of the fluid and also to the modification of the electric field distribution within the oil and paper insulation that is caused by the variation of the permittivities of the liquid and solid insulation when NPs are present.

The improvement is less noticeable for the impulse tests, and the values of the average impulse dielectric strength of NF-impregnated samples are only 2% higher than those of MO-impregnated specimens. More research is needed to determine if higher concentrations of NPs could lead to higher values of the impulse dielectric strength of NF-impregnated cellulose insulation. Additionally, it would be important to repeat the study considering other types of NPS, base fluids, and solid materials.

## Figures and Tables

**Table 1:** Summary of the studies published by different authors on the characterization of the creeping flashover voltage and dielectric strength (DS) of cellulose materials with NPs.

Ref	NPs	Cellulose Material	Tests Performed	Improvement in BDV	Comments
[24]	Al${}_{2}$O${}_{3}$	Pressboard	Creeping flashover AC	7%	-
[24]	Al${}_{2}$O${}_{3}$	Pressboard	Creeping flashover impulse	14% Negative 15% Positive	-
[25]	TiO${}_{2}$	Pressboard	Creeping flashover AC	18.8%	NP sizes 5 to 30 nm
[26]	Fe${}_{3}$O${}_{4}$	Pressboard	Lightning impulse creeping flashover	15–17%	Two distance for electrodes used
[27]	h-BN	Kraft paper	AC DS	2%	Compared fresh and aged samples
[28]	TiO${}_{2}$	Pressboard	DC DS	12.9%	Several NP sizes 5 to 15 nm
[29]	TiO${}_{2}$	NP-doped Kraft paper	AC BDV	20.83%	Several wt% of NP

**Table 2:** Weibull parameters for the AC dielectric strength tests.

Weibull Parameters	MO-Paper	NF-Paper
*a* (kV/mm)	69.2	79.7
*b*	6.5	11.7

**Table 3:** Statistic analysis of the AC dielectric strength tests for both types of specimens.

AC Dielectric Strength	MO-Paper	NF-Paper
Mean (kV/mm)	59.8	75.3
Std dv	11.24	5.47
Fail prob 1% (kV/mm)	34.0	53.7
Fail prob 25% (kV/mm)	57.1	71.6
Fail prob 50% (kV/mm)	65.4	77.2
Fail prob 90% (kV/mm)	78.7	85.6

**Table 4:** Maximum electric field in paper and oil for a testing voltage of 5.5 kV.

	Kraft Paper-MO		Kraft Paper-NF	
	Oil	Paper	Oil	Paper
Relative permittivity	2.2	3.9	3.2	4.9
Maximum E (kV/mm)	114.42	64.95	99.30	64.86

**Table 5:** Impulse test applied with and without breakdown for MO-impregnated samples.

Voltage (kV)	Impulses Applied	Number of Breakdowns
7	3	0
8	15	0
9	18	2
10	11	3
11	8	1
12	1	1

**Table 6:** Impulse test applied with and without breakdown for NF-impregnated samples.

Voltage (kV)	Impulses Applied	Number of Breakdowns
7	3	0
8	18	0
9	12	1
10	6	3
11	3	1
12	1	1

**Table 7:** Weibull parameters for the impulse dielectric strength of MO-impregnated and NF-impregnated paper.

Weibull Parameters	MO-Impregnated Paper	NF-Impregnated Paper
*a* (kV/mm)	127.9	129.8
*b*	10.3	10.2

**Table 8:** Main values obtained from the Weibull fit of the results of the impulse dielectric strength tests for both types of specimens.

Impulse Strength	MO-Impregnated Paper	NF-Impregnated Paper
Mean (kV/mm)	121.8	124.1
Std dv (kV/mm)	7.45	8
Fail prob 1% (kV/mm)	81.9	82.5
Fail prob 25% (kV/mm)	113.4	114.8
Fail prob 50% (kV/mm)	123.4	125.2
Fail prob 90% (kV/mm)	138.7	140.9

**Table 9:** BDV obtained for positive lightning impulse for MO and two NFs with different Fe${}_{3}$O${}_{4}$ concentrations.

	MO	NF 0.2 g/L	NF 0.6 g/L
Impulse BDV (kV)	34.37	35.7	51.46

## Data Availability

Not applicable.
